# Impact of Non-pharmacological Chronic Hypertension on a Transgenic Rat Model of Cerebral Amyloid Angiopathy

**DOI:** 10.3389/fnins.2022.811371

**Published:** 2022-03-15

**Authors:** Aleksandra Stanisavljevic, Joseph M. Schrader, Xiaoyue Zhu, Jennifer M. Mattar, Ashley Hanks, Feng Xu, Mark Majchrzak, John K. Robinson, William E. Van Nostrand

**Affiliations:** ^1^Department of Biomedical and Pharmaceutical Sciences, University of Rhode Island, Kingston, RI, United States; ^2^George and Anne Ryan Institute for Neuroscience, University of Rhode Island, Kingston, RI, United States; ^3^Department of Psychology, University of Rhode Island, Kingston, RI, United States

**Keywords:** cerebral amyloid angiopathy, hypertension, comorbidity, transgenic rat, cerebral microbleeds

## Abstract

Cerebral amyloid angiopathy (CAA), a common comorbidity of Alzheimer’s disease (AD), is a cerebral small vessel disease (CSVD) characterized by deposition of fibrillar amyloid β (Aβ) in blood vessels of the brain and promotes neuroinflammation and vascular cognitive impairment and dementia (VCID). Hypertension, a prominent non-amyloidal CSVD, has been found to increase risk of dementia, but clinical data regarding its effects in CAA patients is controversial. To understand the effects of hypertension on CAA, we bred rTg-DI transgenic rats, a model of CAA, with spontaneously hypertensive, stroke prone (SHR-SP) rats producing bigenic rTg-DI/SHR-SP and non-transgenic SHR-SP littermates. At 7 months (M) of age, cohorts of both rTg-DI/SHR-SP and SHR-SP littermates exhibit elevated systolic blood pressures. However, transgene human amyloid β-protein (Aβ) precursor and Aβ peptide levels, as well as behavioral testing showed no changes between bigenic rTg-DI/SHR-SP and rTg-DI rats. Subsequent cohorts of rats were aged further to 10 M where bigenic rTg-DI/SHR-SP and SHR-SP littermates exhibit elevated systolic and diastolic blood pressures. Vascular amyloid load in hippocampus and thalamus was significantly decreased, whereas pial surface vessel amyloid increased, in bigenic rTg-DI/SHR-SP rats compared to rTg-DI rats suggesting a redistribution of vascular amyloid in bigenic animals. There was activation of both astrocytes and microglia in rTg-DI rats and bigenic rTg-DI/SHR-SP rats not observed in SHR-SP rats indicating that glial activation was likely in response to the presence of vascular amyloid. Thalamic microbleeds were present in both rTg-DI rats and bigenic rTg-DI/SHR-SP rats. Although the number of thalamic small vessel occlusions were not different between rTg-DI and bigenic rTg-DI/SHR-SP rats, a significant difference in occlusion size and distribution in the thalamus was found. Proteomic analysis of cortical tissue indicated that bigenic rTg-DI/SHR-SP rats largely adopt features of the rTg-DI rats with enhancement of certain changes. Our findings indicate that at 10 M of age non-pharmacological hypertension in rTg-DI rats causes a redistribution of vascular amyloid and significantly alters the size and distribution of thalamic occluded vessels. In addition, our findings indicate that bigenic rTg-DI/SHR-SP rats provide a non-pharmacological model to further study hypertension and CAA as co-morbidities for CSVD and VCID.

## Introduction

Cerebral small vessel diseases (CSVD) occur in many elderly patients, cause vascular cognitive impairment and dementia (VCID) and are contributors to ischemic and hemorrhagic strokes (Shi and Wardlaw, 2016; Mustapha et al., 2019; Pasi and Cordonnier, 2020). CSVD and VCID are growing concerns as the elderly population increases. Two general classes of CSVD are non-amyloidal CSVD and amyloidal CSVD. Non-amyloidal CSVD include lifestyle induced diseases such as hypertension (Cuadrado-Godia et al., 2018) and arteriolosclerosis (Li et al., 2018), but also hereditary diseases such as cerebral autosomal dominant arteriopathy with sub-cortical infarcts and leukoencephalopathy (CADASIL) (Mancuso et al., 2020). Cerebral amyloid angiopathy (CAA), an example of amyloidal CSVD, is a prevalent condition in the elderly and is a common comorbidity of Alzheimer’s disease (AD) (Gilbert and Vinters, 1983; Thal et al., 2002; Viswanathan and Greenberg, 2011; Mehndiratta et al., 2012). CAA is characterized by deposition of fibrillar amyloid β-protein (Aβ) in blood vessels of the brain, including capillaries, arterioles and small arteries of the cortex and meninges (Auriel and Greenberg, 2012; Denver et al., 2019). Patients present with a decline in cognition as CAA contributes to development of VCID (Helman, 2018). CAA affects ≈80% of individuals over the age of 65 years (Boyle et al., 2015) and is present to some extent in nearly all AD patients (Ellis et al., 1996). Though the elderly are mainly affected by sporadic forms (Biffi and Greenberg, 2011), familial forms causing early onset of the disease also exist. Familial forms arise from mutations in the amyloid precursor protein (AβPP) gene that change the Aβ peptide sequence (Levy et al., 1990; Van Broeckhoven et al., 1990; Tagliavini et al., 1999; Grabowski et al., 2001; Biffi and Greenberg, 2011). These changes in the Aβ peptides appear to alter their biophysical properties and enhance fibrillogenesis (Van Nostrand et al., 2001; Melchor et al., 2008).

CAA exists in two types: 1 and 2. CAA type-2 is defined by amyloid deposits within the vessel wall and typically does not elicit a robust perivascular inflammatory response in the absence of amyloid infiltrating the surrounding brain parenchyma (Thal et al., 2002; Attems et al., 2011; Davis et al., 2018). In contrast, CAA type-1 amyloid is found along capillary vessel walls, allowing for interaction with the surrounding parenchymal tissue and induces a strong perivascular neuroinflammatory response (Thal et al., 2002; Attems et al., 2011).

Although CAA is recognized as a cause of intracerebral hemorrhagic strokes (ICH) in normotensive elderly patients (Auriel and Greenberg, 2012), hypertension (HTN), a non-amyloidal form of CSVD, is the most common cause of ICH (Broderick et al., 2020). Nearly 70% of individuals over the age of 65 in the United States are diagnosed with HTN (Mozaffarian et al., 2015), which is a common risk factor for dementia (Welsh et al., 2014). Despite their separate risks for ICH, it remains unclear how CAA and HTN potentially interact to impact ICH and VCID. For example, in a comparison of CAA patients and HTN patients that had ICH it was found that some differences between these two CSVDs exist, but it is still difficult to distinguish the two (Zhan et al., 2004).

Previously, we described the generation and characterization of a new transgenic rat model of CAA type-1, rTg-DI, that faithfully recapitulates many pathological features of the disease in humans (Davis et al., 2018). The rTg-DI model utilizes the expression of human AβPP in brain harboring two familial CAA mutations of Aβ, the Dutch (E22Q) and Iowa (D23N) mutations. rTg-DI rats develop early onset and progressive accumulation of CAA type-1 pathologies. Vascular amyloid first appears at ≈3 months with a subsequent emergence of behavioral deficits, perivascular neuroinflammation, microbleeds, small vessel occlusions and progressive loss of white matter (Zhu et al., 2020; Lee et al., 2021). These findings indicate that rTg-DI rats provide a useful preclinical platform to investigate the pathogenesis of CAA and microbleeds.

On the other hand, the spontaneously hypertensive stroke-prone (SHR-SP) rat is commonly used to investigate HTN and ICH. This particular model was derived by selective in-breeding of animals that present elevated blood pressure and HTN. SHR-SP rats also present with spontaneous strokes (Kimura et al., 2000). The HTN phenotype resulting from this breeding is due to multiple genetic factors that remain undefined; phenotyping rather than genotyping is therefore required. The SHR-SP rat provides a model that spontaneously develops HTN, is a more clinically relevant model and eliminates any undesired and potentially confounding side effects that could be introduced using pharmacological-induced HTN. In the present study, we bred rTg-DI rats with SHR-SP rats to generate a model of emerging CAA on a hypertensive background to investigate how the two distinct CSVDs interact to affect CAA progression and thrombotic vascular events.

## Materials and Methods

### Animals

The goal of our study was to evaluate the impacts of chronic, non-pharmacological HTN on the development of CAA and related pathologies in experimental rats. To accomplish this, we used the CAA transgenic rat line (rTg-DI), which is a model of early onset and robust cerebral microvascular amyloid as previously described (Davis et al., 2018). rTg-DI rats, generated on a Sprague-Dawley background, express low levels of human Swedish/Dutch/Iowa mutant AβPP under the control of the neuronal-specific Thy1.2 promoter and results in production of chimeric Dutch/Iowa CAA mutant Aβ peptides in the brain. Aβ accumulation and perivascular inflammation begin at ≈3 months of age and increase in a time dependent manner. Microbleeds and small vessel occlusions emerge at ≈6 months of age and become more numerous with advancing age.

For a model of chronic, non-pharmacological HTN, we used Spontaneously Hypertensive—Stroke Prone (SHR-SP) rats that were obtained from Charles River Laboratories (Kingston, NY). The SHR-SP model was derived from the spontaneously hypertensive (SHR) rat by inbreeding (Okamoto and Aoki, 1963). Phenotypes have been found to be autosomal dominant (Gratton et al., 1998) and can be identified by phenotyping rather than genotyping.

In the present study, rTg-DI rats and rTg-DI negative (WT) littermates served as CAA and controls. To obtain HTN CAA rats, SHR-SP rats were bred with heterozygous rTg-DI rats to produce bigenic rTg-DI/SHR-SP and rTg-DI transgene negative SHR-SP offspring. The rTg-DI/SHR-SP rats used in this study were backcrossed for 5 generational breedings with SHR-SP rats resulting in bigenic rats that possessed the human AβPP transgene and were > 95% on the hypertensive SHR-SP background. rTg-DI negative SHR-SP littermates served as HTN controls. Cohorts of each group of rats were aged to 7 or 10 M.

All rats were housed in a controlled room (22 ± 2°C and 40–60% humidity) on a standard 12 h light cycle. Rat chow and water were available *ad libitum*. All work with animals was approved by the University of Rhode Island Institutional Animal Care and Use Committee and in accordance with the United States Public Health Service’s Policy on Humane Care and Use of Laboratory Animals and was and in compliance with the ARRIVE guidelines.

### Blood Pressure Measurements

Arterial blood pressures were measured using the CODA Non-invasive Blood Pressure System (Kent Scientific, Torrington, CT, United States). Rats were handled and acclimatized to the system for 15 min per day for 5 days before baseline measurements. Before starting, the infrared warming platform was warmed to a constant temperature between 33 and 38°C and maintained for all measurements. Rats were assigned appropriately sized restrainers with nose cones in which excess movement was limited and comfortable respiration was possible. The animals were encouraged to enter the restrainer with minimal guidance. While animals were in the restrainers and placed on the warming platform, tail occlusion cuffs were placed around the caudal region of the tails. The occlusion cuff size was determined based on tail thickness and ability to freely move up and down on the tail. The Volume Pressure Recording Sensor was placed on the tail about 2 mm distal to the occlusion cuff. This sensor was placed so that it could move freely along the tail and inflate enough to impede tail blood flow. Animal body temperatures were measured after 5 min of acclimatization in the restrainer and on the warming platform and ensured to read between 33 and 38°C. Tail temperatures were monitored until 32–35°C was maintained. Once body and tail temperatures were constant, systolic and diastolic blood pressures were measured automatically using the default settings for blood pressure monitoring on the blood pressure system. The averages of systolic and diastolic readings were calculated by the system.

### Behavioral Testing

Open Field: To assess general exploration behavior, mobility and health the younger group of rats were subjected to Open Field testing at 7 M. After being habituated to the room for 30 min, animals were placed in the center of a 92 cm^2^ field (Stoelting Co., Wood Dale, IL), inside a semi-opaque cylinder and held for 20 s to acclimate to the apparatus. After acclimation, animals were allowed to explore the open field for a total of 5 min. Total distance traveled for each animal was tracked using AnyMaze™ tracking software (Stoelting Co., Wood Dale, IL) and the number of rearings were scored via manual keypress by the experimenter.

Rotarod: the rats were habituated to the test room for 30 min and then pre-trained by first being able to balance on the stationary rod for 10 s and then 60 s on a rod rotating at a speed of 5 rpm. For the testing protocol, the rod accelerated from 5 to 40 rpm in a period of up to 300 s or until the animal fell off the rod. The rats were returned to their home cages between trials for a total of three trials with 15 min ITIs. The mean latency to fall of the three trials is used to compare time spent on the rod.

Unreinforced Radial Arm Maze (URAM): This procedure followed an unrewarded version of the Radial Arm Maze (RAM) task where none of the arms were reinforced. At the start of each trial, rats were placed in the center circle of an 8-armed RAM apparatus (Stoelting Co., Wood Dale, IL). Arm entries were manually recorded by the experimenter and entry was defined as all four paws entered into the shaft of one arm. Trials terminated after 5 min or when the rat had successfully visited each arm of the apparatus once. In this configuration, this task reveals a slowed rate of arm entrances thought to reflect sensory-motor slowing in the rTg-DI rats as previously reported (Popescu et al., 2020).

### Brain Collection and Tissue Preparation

Rats were injected with 1 mL/kg of ketamine and 0.5 mL/kg xylazine for deep anesthesia. The chest cavity was opened for intracardial perfusion with 1 M phosphate buffered saline (PBS) containing 0.05% heparin at 20 mL/min perfusion rate for 15 min. Forebrains were removed and cut in half along the mid-sagittal line. The right hemisphere was immediately embedded in Tissue-Plus OCT Compound (Fisher Healthcare, Houston, TX), frozen on dry ice, and stored in −80°C. These tissues were later sectioned on the sagittal plane at 20 μm thickness, mounted on Colorfrost/Plus slides (Thermo Fisher Scientific, Houston, TX) and stored at −80°C. In other cases, brain sections were collected and lysed with radioimmunoprecipitation assay (RIPA) buffer via 12 × 1 s bursts of sonication on ice followed by a 1 h incubation on ice. Samples were then normalized using BCA protein assay Kit (Thermo Fisher Scientific, Houston, TX).

The left hemisphere was immersion -fixed with 4% paraformaldehyde (PFA) for 24 h and then immersed in 30% sucrose in PBS for 48 h for cryoprotection. These tissues were then embedded in OCT Compound and frozen at −80°C. PFA fixed tissue was sagittal cut at 20 μm thickness using the Leica CM 3050S Cryostat (Leica Microsystems Inc., Buffalo Grove, IL), placed in a flotation PBS bath at −16°C, and then mounted on Colorfrost/Plus slides (Thermo Fisher Scientific, Houston, TX) coated using EMS Tissue Capture Pen (Electron Microscopy Sciences, Hatfield, PA) and stored at −80°C.

### Immunoblot Quantitation of AβPP

The levels of AβPP in lysed forebrain tissue sections were determined by performing quantitative immunoblotting as described (Davis et al., 2004). Samples were probed with horseradish peroxidase (HRP) labeled-monoclonal antibody P2-1 (specific for human AβPP) (Suzukit et al., 1989) at a concentration of 1:1,000 overnight. HRP-catalyzed chemiluminescent signal was revealed using SuperSignalWest Femto Maximum Sensitivity Substrate (Pierce Biotechnology, Rockford, IL, Thermo Fisher Scientific cat# 34096) and chemiluminescent signal was detected and quantified using an Odyssey Fc imager (LI-COR, Lincoln, NE).

### Quantitation of Aβ Peptides

Total Aβ40 and Aβ42 levels were determined by ELISA of guanidine lysates of rat forebrain tissue. In the sandwich ELISAs, Aβ40 and Aβ42 were captured using their respective carboxyl-terminal specific antibodies mAb2G3 and mAb21F12 and biotinylated m3D6, specific for human Aβ, was used for detection (Johnson-Wood et al., 1997; DeMattos et al., 2002). All lysates were measured in triplicates and compared to linear standard curves of known concentrations of human Aβ using a Spectramax M2 plate reader (Molecular Devices, Sunnyvale, CA).

### Immunohistochemical Analysis

Antigen retrieval was performed by treating the tissue sections with proteinase K (0.2 mg/ml) for 10 min at 22°C. Tissue sections were then blocked in Superblock blocking buffer (cat. #37518, Thermo Fisher Scientific, Franklin, MA) containing 0.3% Triton X-100 at room temperature for 30 min and incubated with individual primary antibodies at the following dilutions overnight: rabbit polyclonal antibody to collagen IV to identify cerebral blood vessels (1:200, SD2365885, Invitrogen); goat polyclonal antibodies to glial fibrillary acidic protein (GFAP) to detect astrocytes (1:250, ab53554, Abcam) and ionized calcium-binding adapter molecule 1 (Iba-1) to detect microglia (1:250, NB100-1028, Novus). The primary antibodies were detected with Alexa Fluorescent 594- or 488-conjugated secondary antibodies (1:1,000). Staining for fibrillar amyloid was performed using thioflavin S (123H0598, Sigma-Aldrich). Prussian blue iron staining was performed to detect hemosiderin deposits reflecting signs of previous microbleeds (Gomori, 1936; Davis et al., 2018). Von Kossa calcium staining was used to detect small vessel calcified occlusion in the brain (Rungby et al., 1993; Davis et al., 2018).

### Quantitative Measures of Cerebral Vascular Pathologies

The percent area amyloid coverage of cerebral microvessels, the number of microbleeds and the number of occluded/calcified vessels in the cortex, hippocampus and thalamus were determined in the rats using stereological principles as in previously described studies (Long et al., 1998; Davis et al., 2018). Diameters of vessel occlusions and thalamic area occupied by occlusions were measured. Diameters were measured using the “point to point function” and the free hand area function was used to delineate an area around the outermost occlusions in the analyzed section. Vessel occlusion diameters were categorized by size: < 30 μm (small) or > 30 μm (large). The number of occlusions in each size was reported as a percent of the total number of occlusions. The percent area CAA coverage was determined as the percent area of thioflavin stain overlapping with collagen IV stain. All images were collected with the Keyence BZ-X710 Microscope (RRID:SCR_017202) and analyzed with the Keyence BZ-X Analyzer Software Version 1.3.1.1 (Keyence Corp. Osaka, Japan). The number of microbleeds and occluded vessels were measured using with Image J Software Version 1.52a (ImageJ, RRID:SCR_003070, National Institute of Health, United States).

### Protein Digest of Cortical Tissue

The cortical region from brain sections of WT, SHR-SP, rTg-DI and bigenic rTg-DI/SHR-SP rats was collected using laser capture microdissection (LCM). Tissue lysis and sample preparation for MS analysis was performed as previously described (Schrader et al., 2021). Briefly, tissue lysis was achieved via sonication in RIPA buffer. 25 μL dithiothreitol (100 mM) was added for protein denaturation with incubation and shaking (300 rpm) at 95°C for 15 min. Proteins were alkylated by incubation in the dark with 25 μL iodoacetamide (200 mM) 30 min at 20°C, and then precipitated and concentrated via chloroform-methanol-water (1:2:1) precipitation. Proteins were resuspended in sodium deoxycholate (DOC) (3% w/v in 50 mM ammonium bicarbonate) and digested with TPCK-treated trypsin (Sciex, Framingham, MA), in a barocycler (Pressure Biosciences Inc, Easton MA) as previously described (Schrader et al., 2021). DOC was precipitated by addition of formic acid and centrifugation as previously described, and the supernatant was collected for LC-MS/MS analysis.

### Analysis by LC-QTOF/MS

A SCIEX 5600 TripleTOF mass spectrometer, operated in positive ion mode using a DuoSpray™ ion source (AB Sciex, Concord, Canada), coupled to an Acquity UPLC HClass system (Waters Corp., Milford, MA, United States) for chromatographic separation as was used for all proteomic experiments as previously described (Schrader et al., 2021). An Acquity VanGuard pre-column (2.1 × 5 mm, 300 Å, 1.7 μm) preceding an Acquity UPLC Peptide BEH C18 (2.1 × 150 mm, 300 Å, 1.7 μm) column were used for peptide separation according to the previously described method (Schrader et al., 2021). TOF calibration was monitored by injection of trypsin-digested β-galactosidase every 4 samples. Analyst TF 1.7.1 software (AB Sciex, Concord, Canada) in data-independent acquisition (DIA) mode was used for data acquisition. All mass spec settings were exactly as previously described (Schrader et al., 2021).

### Data Processing

Spectronaut (Biognosys, Schlieren, Switzerland), referencing our previously formed spectral library (Schrader et al., 2021) was used for all protein identification and quantification. Factory defaults were used for all Spectronaut settings, except “used Biognosys’ iRT kit” and “PTM localization” were deselected. Spectronaut protein intensities were converted to molar concentrations (pmol/mg brain tissue) using the total protein approach (TPA) (Wiśniewski and Rakus, 2014). A baseline concentration (0.03 pmol/mg tissue) was imputed for concentrations of 0 in individual samples as previously described (Schrader et al., 2021). Threshold cutoffs of ≥ 50% increase or ≥ 30% decrease to identify differentially expressed proteins, and Student’s *t*-test was used to determine statistical significance (*P* ≤ 0.05) as previously described (Schrader et al., 2021).

### Statistical Analysis

For blood pressure readings, ordinary one- way ANOVA, followed by an uncorrected Fisher’s test LSD, with single pooled variance compared the means of each group. For behavioral data, it was noted that there was large and differing variance in the groups, therefore Welch’s correction was used for ANOVA and individual *t*-test. Unpaired two tailed *t*-tests were completed for ELISAs of total Aβ and for CAA load. For number of occluded vessel and number of microbleeds, it was observed that the data was not normally distributed, therefore a non-parametric was indicated. For these data, non- parametric one-way ANOVA Kruskal Wallis test followed by Dunn’s multiple comparisons test was completed. Unpaired, one tailed, parametric *t*-tests were used to compare percentages of vessel occlusions within the listed size ranges. A non-parametric, two tailed Mann-Whitney test was employed to compare percent area coverage of vessel occlusions in thalami. GraphPad Prism Version 9.1.2 was used for all statistical analyses.

## Results

### Hypertension Phenotype Is Preserved in 7 Month Bigenic Rat Model of Cerebral Amyloid Angiopathy/Spontaneously Hypertensive, Stroke Prone Rats

**FIGURE 1:**
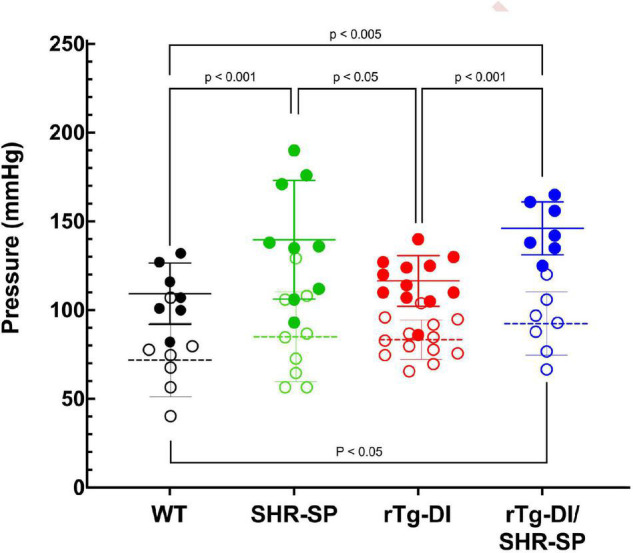
Bigenic rTg-DI rats exhibit elevated blood systolic pressure at 7 M of age. Systolic (closed circles) and diastolic (open circles) blood pressure readings were measured in WT (black; *n* = 7), SHR-SP (green; *n* = 9), rTg-DI (red; *n* = 12), and bigenic rTg-DI/SHR-SP (blue; *n* = 7) rats at 7 M of age. Mean pressures are represented by solid or hashed horizontal lines ± SD. The systolic blood pressures of SHR-SP and rTg-DI/SHR-SP rats are significantly increased from those of WT and rTgDI rats. One-way ANOVA shows *P* < 0.001 between SHR-SP and WT, *P* < 0.05 between SHR-SP and rTg-DI, *P* < 0.005 between bigenic rTg-DI/SHR-SP and WT, and *P* < 0.001 between bigenic rTg-DI/SHR-SP and rTg-DI. These results indicate that increased systolic blood pressure is preserved with addition of the rTg-DI transgene on the SHR-SP background.

Groups of rTg-DI, SHR-SP, bigenic rTg-DI/SHR-SP and WT rats (*n* = 7) were aged to 7 months (M). Systolic and diastolic blood pressure readings were acquired for each rat of the four strains as shown in Figure 1. Data analysis for systolic blood pressures was completed with one-way ANOVA having no matching or pairing with single pooled variance, comparing means of each group and an uncorrected Fisher’s test LSD (*F* = 5.418, *p* = 0.0041, *R*^2^ = 0.3440). Systolic blood pressure readings were significantly increased in SHR-SP and bigenic rTg-DI/SHR-SP rats compared to control groups of WT and rTg-DI rats. Systolic blood pressures of SHR-SP animals were significantly different from those of WT and rTg-DI animals (*P* < 0.01 and *P* < 0.05, respectively). Bigenic rTg-DI/SHR-SP systolic blood pressures were also increased compared to WT and rTg-DI (*P* < 0.005 and *P* < 0.01, respectively) as indicated by one-way ANOVA. Data analysis for diastolic blood pressures was also completed with one-way ANOVA having no matching or pairing with single pooled variance, comparing means of each group and an uncorrected Fisher’s test LSD (*F* = 1.454, *p* = 0.2456, *R*^2^ = 0.1199). Differences in diastolic blood pressures were only observed between rTg-DI/SHR-SP and WT animals (*P* < 0.05). These results indicate that systolic blood pressure is increased in SHR-SP animals and that this increase is maintained with addition of the rTg-DI transgene on the SHR-SP background.

### Spontaneously Hypertensive, Stroke Prone Background Does Not Influence Human AβPP Transgene Expression

**FIGURE 2:**
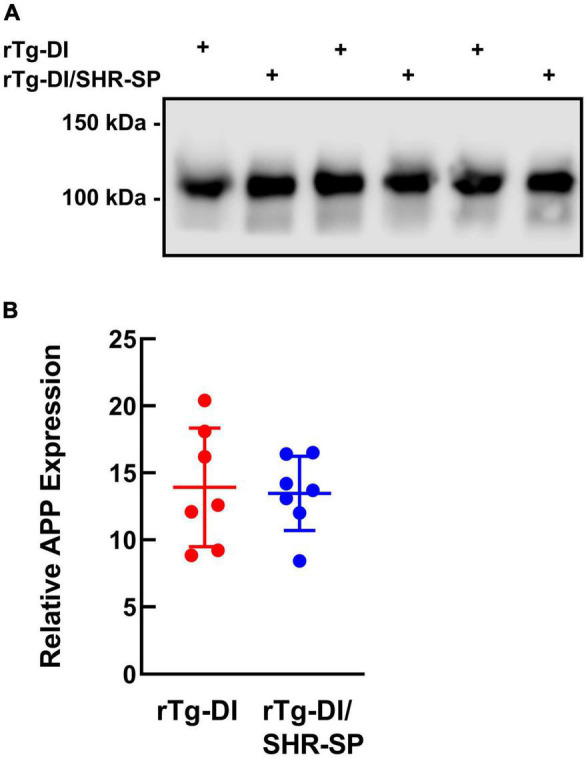
Equivalent transgenic human AβPP expression in rTg-DI and bigenic rTg-DI/SHR-SP rats. **(A)** Representative immunoblots of human AβPP expression in total brain homogenates from 7M rTg-DI rats and bigenic rTg-DI/SHR-SP rats. **(B)** Quantitative immunoblotting was performed to measure human AβPP in brain homogenates of rTg-DI rats (red circles) and bigenic rTg-DI/SHR-SP rats (blue circles). The data presented are the means ± S.D. of *n* = 7 rats per each group. Unpaired *t*-test showed no differences in human AβPP expression in 7 M rTg-DI and bigenic rTg-DI/SHR-SP animals.

The levels of AβPP expression in rTg-DI and bigenic rTg-DI/SHR-SP rats were measured using quantitative immunoblotting (Figure 2). Unpaired *t*-test shows that there are no differences in human AβPP protein levels between rTg-DI and bigenic rTg-DI/SHR-SP animals. This indicates equivalent expression of AβPP in both rTg-DI and bigenic rTg-DI/SHR-SP rats and preservation of the rTg-DI phenotype on the SHR-SP background.

### Hypertension Phenotype Does Not Affect Brain Accumulation of Ab40 and Ab42 in 7 M Bigneic Rat Model of Cerebral Amyloid Angiopathy/Spontaneously Hypertensive, Stroke Prone Rats

**FIGURE 3:**
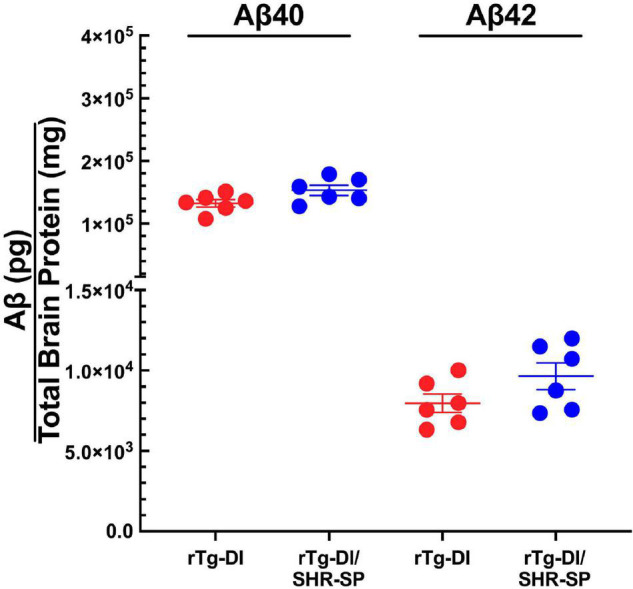
Equivalent Aβ levels in 7 M rTg-DI and bigenic rTg-DI/SHR-SP rats. The levels of total Aβ40 and Aβ42 peptides in the forebrains of rTg-DI rats (red circles) and bigenic rTg-DI/SHR-SP rats (blue circles) were measured by ELISA as described under section “Materials and Methods.” The data presented are the means ± S.D. of *n* = 7 rats per group. Unpaired *t*-test showed that there are no significant differences between Aβ40 and Aβ42 levels in rTg-DI and bigenic rTg-DI/SHR-SP animals.

We next measured the levels of total Aβ40 and total Aβ42 in rTg-DI and bigenic rTg-DI/SHR-SP rats using specific ELISA assays. Analysis of the data presented in Figure 3 using unpaired, two tailed *t*-test showed that there were no differences between total Aβ40 and Aβ42 levels in rTg-DI and bigenic rTg-DI/SHR-SP animals. These findings indicate that the hypertensive SHR-SP background does not influence the accumulation of Aβ peptides in the rTg-DI model at 7 M of age.

### Hypertension Phenotype Does Not Influence Behavioral Deficits in Bigenic Rat Model of Cerebral Amyloid Angiopathy/Spontaneously Hypertensive, Stroke Prone Rats at 7 M

**FIGURE 4:**
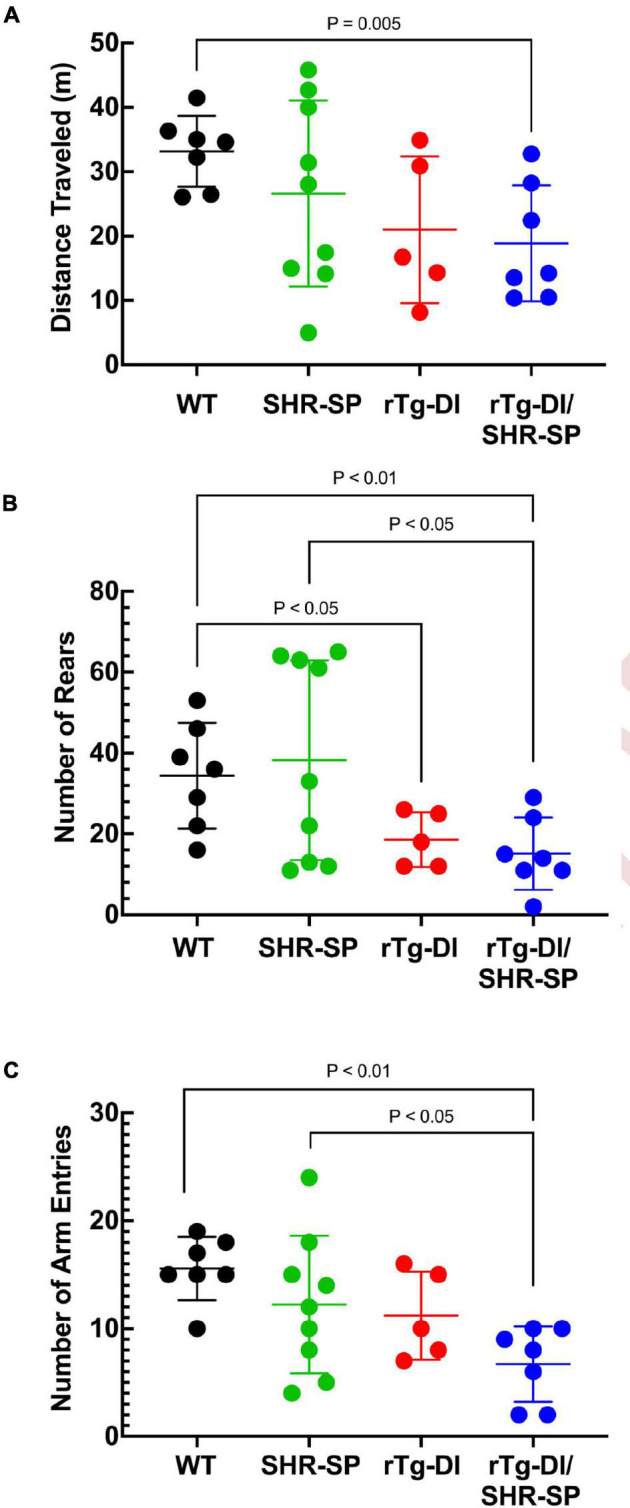
Behavioral deficits of 7 M rTg-DI and bigenic rTg-DI/SHR-SP rats. Bigenic rTg-DI/SHR-SP rats were not significantly different than rTg-DI rats in the number of rears **(A)** or distance traveled **(B)** in the open field. Similarly, no significant differences were found between rTg-DI and bigenic rTg-DI/SHR-SP animals in the number of arm entries **(C)** in Unreinforced Radial Arm Maze (URAM). WT rats (black; *n* = 7), SHR-SP (green; *n* = 9), rTg-DI (red; *n* = 5), and bigenic rTg-DI/SHR-SP (blue; *n* = 7). Data presented are the mean ± SD. These data show that the hypertensive SHR-SP background does not significantly impact behavioral deficits of rTg-DI rats at 7 M of age.

rTg-DI rats were previously shown to exhibit a “sensory-motor slowing” phenotype with the onset of microvascular amyloid accumulation (Popescu et al., 2020). This phenotype was seen by comparing the 7 M WT vs. rTg-DI animal rearing measures in the open field (Figure 4A) and is emerging in the open field distance traveled (Figure 4B) and in the number of arm entries measure in the unreinforced radial arm maze (Figure 4C). No significant differences in distance traveled in open field or number of rears were seen between rTg-DI and bigenic rTg-DI/SHR-SP rats in any of the behavioral measures, indicating that most of the difference between the bigenic rats and SHR-SP rats is contributed by the rTg-DI genotype. All groups also performed similarly in rotarod task, indicating that differences in other assays were not due to motor impairment (data not shown). These findings indicate that non-pharmacological, chronic HTN does not alter the behavioral deficits observed in 7 M old rTg-DI rats.

### Hypertension Phenotype Is Preserved in 10 M Bigenic Rat Model of Cerebral Amyloid Angiopathy/Spontaneously Hypertensive, Stroke Prone Rats

**FIGURE 5:**
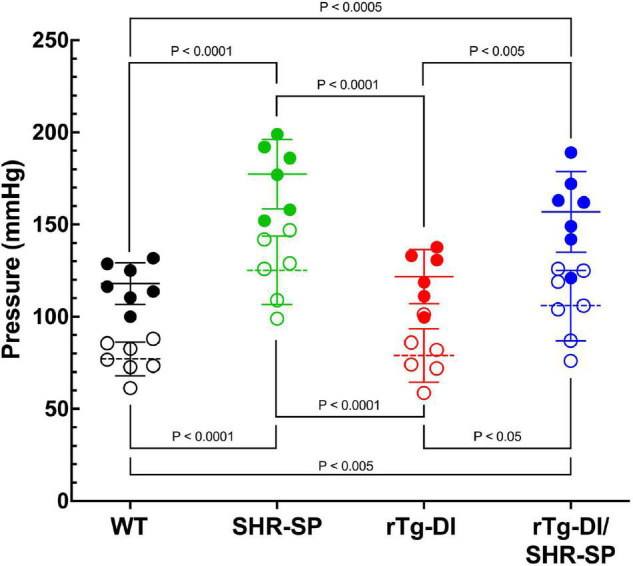
Bigenic rTg-DI rats exhibit elevated systolic and diastolic blood pressure at 10 M of age. Systolic (closed circles) and diastolic (open circles) blood pressure readings were measured in WT (black; *n* = 7), SHR-SP (green; *n* = 6), rTg-DI (red; *n* = 6), and bigenic rTg-DI/SHR-SP (blue; *n* = 7) rats at 10 M. Mean pressures are represented by solid or hashed horizontal lines ± SD. The systolic blood pressures of SHR-SP and bigenic rTg-DI/SHR-SP rats are significantly increased from those of WT and rTg-DI rats. It is shown by one-way ANOVA that *P* < 0.0001 for SHR-SP compared to both WT and rTg-DI, *P* < 0.0005 for bigenic rTg-DI/SHR-SP compared to WT and *P* < 0.005 compared to rTg-DI. The same differences between groups are shown in diastolic pressures by one-way ANOVA. Comparisons of SHR-SP to WT and rTg-DI both are *P* < 0.0001. Diastolic pressures of bigenic rTg-DI/SHR-SP compared to those of WT and rTg-DI are *P* < 0.005 and *P* < 0.05, respectively. These results indicate that the increase in both systolic and diastolic blood pressures is maintained with addition of the rTg-DI transgene on the SHR-SP background.

Since no profound changes were observed in 7 M bigenic rTg-DI/SHR-SP rats, a second cohort of rTg-DI, SHR-SP, and bigenic rTg-DI/SHR-SP and WT rats were aged further to 10 M. Systolic and diastolic blood pressure readings were acquired for each rat of the four strains as shown in Figure 5. Data analysis for systolic blood pressures was completed with one-way ANOVA having no matching or pairing with single pooled variance, comparing means of each group and an uncorrected Fisher’s test LSD (*F* = 17.51, *P* < 0.0001, *R*^2^ = 0.7048). Systolic blood pressure readings were significantly increased in SHR-SP and bigenic rTg-DI/SHR-SP rats compared to control groups of WT and rTg-DI rats. Systolic blood pressures of SHR-SP animals were significantly different from those of WT and rTg-DI animals (both comparisons *P* < 0.0001). Bigenic rTg-DI/SHR-SP systolic blood pressures were similarly increased compared to WT and rTg-DI (*P* < 0.0005 and *P* < 0.005, respectively). Data analysis for diastolic blood pressures was completed in the same manner by one-way ANOVA having no matching or pairing with single pooled variance, comparing means of each group and an uncorrected Fisher’s test LSD (*F* = 13.53, *P* = < 0.0001, *R*^2^ = 0.6485). Similar to the systolic readings, diastolic blood pressure readings were significantly increased in SHR-SP and rTg-DI/SHR-SP rats compared to both control groups WT and rTg-DI. Diastolic pressures of SHR-SP animals were significantly increased from those of WT and rTg-DI animals (*P* < 0.0001 for both comparisons). Bigenic rTg-DI/SHR-SP diastolic blood pressures were also increased compared to WT and rTg-DI by (*p* < 0.0005 and *p* < 0.05, respectively). No differences were observed between SHR-SP and bigenic animals in either blood pressure reading. These results indicate that systolic and diastolic blood pressures are both increased in 10 M SHR-SP animals and that this increase is maintained with addition of the rTg-DI transgene in the bigenic animals.

### Hypertension Phenotype Does Not Influence the Brain Accumulation of Aβ40 and Ab42 in 10 M Bigenic Rat Model of Cerebral Amyloid Angiopathy/Spontaneously Hypertensive, Stroke Prone Rats

**FIGURE 6:**
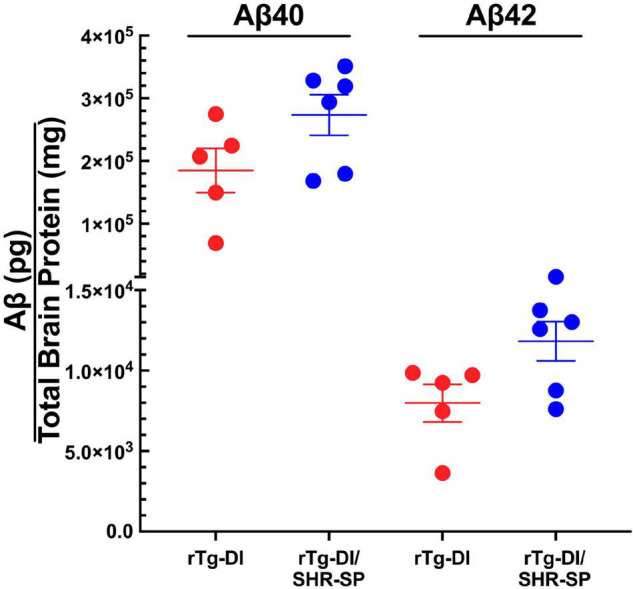
Similar Aβ levels in 10 M rTg-DI and bigenic rTg-DI/SHR-SP rats. The levels of total Aβ40 and Aβ42 peptides in the forebrains of rTg-DI rats (red circles) and bigenic rTg-DI/SHR-SP rats (blue circles) were measured by ELISA as described under section “Materials and Methods.” The data presented are the means ± S.D. of *n* = 5 for rTg-DI rats and *n* = 6 for bigenic rTg-DI/SHR-SP rats. Unpaired *t*-test showed that there are no significant differences between Aβ40 and Aβ42 levels in rTg-DI and rTg-DI/SHR-SP animals.

We next measured the levels of total Aβ40 and total Aβ42 in 10 M rTg-DI and bigenic rTg-DI/SHR-SP rats using previously described ELISA assays. Analysis of the data presented in Figure 6 using unpaired, two tailed *t*-test shows that, similar to the 7 M old rats, there were no differences between total Aβ40 and Aβ42 levels in rTg-DI and bigenic rTg-DI/SHR-SP animals. These findings indicate that the hypertensive SHR-SP background does not influence accumulation of Aβ peptides in the rTg-DI rats as they age further to 10 M.

### Hypertension Phenotype Alters the Distribution of Cerebral Amyloid Angiopathy in 10 M Bigenic Rat Model of Cerebral Amyloid Angiopathy/Spontaneously Hypertensive, Stroke Prone Rats

**FIGURE 7:**
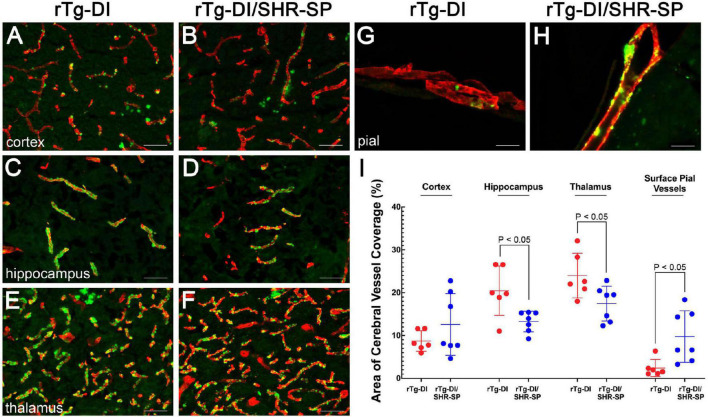
CAA loads in 10 M rTg-DI rats and bigenic rTg-DI/SHR-SP rats. Representative images of brain sections from 10 M old rTg-DI rats **(A,C,E,G)** and bigenic rTg-DI/SHR-SP rats **(B,D,F,H)** were stained for fibrillar amyloid using thioflavin-S (green) and immunolabeled for collagen type IV to identify cerebral vessels (red). Scale bars = 50 μM. **(I)** Quantitation of thioflavin-S positive vascular amyloid load in different brain regions of rTg-DI rats (red circles) and bigenic rTg-DI/SHR-SP rats (blue circles). Data shown are mean ± S.D of *n* = 6–7 rats per group. Unpaired, two tailed *t*-tests show a significant decrease in CAA load in the hippocampus and thalamus of bigenic rTg-DI/SHR-SP rats compared to that of rTg-DI, both comparisons with *p*-values of *P* < 0.05. Contrarily, surface pial vessel coverage is significantly increased bigenic rTg-DI/SHR-SP rats compared to rTg-DI where *P* < 0.05. These data indicate a change in CAA load in when introducing the rTg-DI transgene onto the SHR-SP hypertensive background.

Although the SHR-SP background does not significantly alter the expression of human AβPP or the accumulation of Aβ peptides, we next evaluated if the hypertensive background influences the amount or distribution of vascular amyloid in 10 M rTg-DI rats. Figure 7 shows representative images of thioflavin S-positive cerebral microvascular amyloid present in the cortex, hippocampus and thalamus, regions that were previously shown to accumulate robust levels of CAA type-1 (Davis et al., 2004; Zhu et al., 2020). In addition, representative images of surface pial vessels are also shown. The percent area of vessel coverage with amyloid in each area was measured for each rat. Unpaired, two tailed *t*-tests were completed for rTg-DI (*n* = 6) and bigenic rTg-DI/SHR-SP (*n* = 7) in each area of interest: cortex, hippocampus, thalamus, and surface pial vessels (Figure 7I). There were significant reductions (*P* < 0.05) in the amount of microvascular amyloid in the hippocampus and thalamus of rTg-DI/SHR-SP rats compared to rTg-DI rats. On the other hand, surface pial vessel coverage was significantly increased (*P* < 0.05) in bigenic rTg-DI/SHR-SP compared to rTg-DI. No thioflavin S-positive CAA was observed in any of the WT or SHR-SP rats (data not shown). These findings suggest that at 10 M of age the hypertensive SHR-SP background causes changes in the amount of vascular amyloid in the hippocampus, thalamus, and surface pial vessels.

### Hypertension Phenotype Does Not Alter Thalamic Glial Activation in 10 M Bigenic Rat Model of Cerebral Amyloid Angiopathy/Spontaneously Hypertensive, Stroke Prone Rats

**FIGURE 8:**
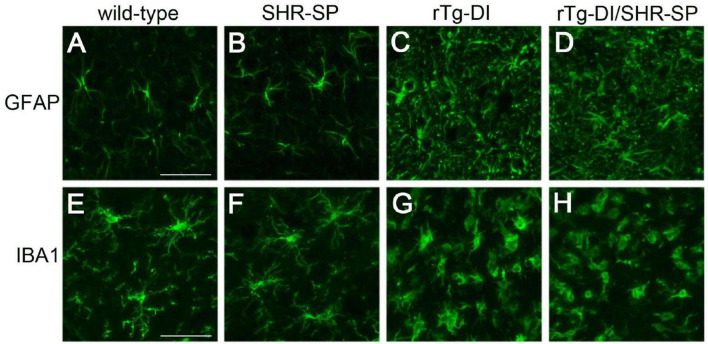
Increased thalamic activated glial cells in 10 M rTg-DI rats and bigenic rTg-DI/SHR-SP rats. Representative images of the thalamus from rat brain sections immunolabeled for GFAP to identify astrocytes (upper panels) and IBA-1 to identify microglia (lower panels). WT rats **(A,E)**, SHR-SP rats **(B,F)**, rTg-DI rats **(C,G)**, and bigenic rTg-DI/SHR-SP rats **(D,H)**. Scale bars = 50 μm. Both rTg-DI and bigenic rTg-DI/SHR-SP rats exhibit increased numbers and activation of glial cells in the thalamus.

In rTg-DI rats, the presence of cerebral microvascular amyloid drives strong neuroinflammation indicated by a marked elevation of reactive perivascular glial cells (Davis et al., 2018; Zhu et al., 2020; Schrader et al., 2021). To determine if the SHR-SP hypertensive background influences this response in rTg-DI rats we performed immunolabeling studies for astrocytes and microglia. Figure 8 shows that both rTg-DI rats and bigenic rTg-DI/SHR-SP rats exhibit a robust increase in thalamic astrocytes compared to WT rats and SHR-SP rats (Figures 8A–D). Similarly, both rTg-DI rats and bigenic rTg-DI/SHR-SP rats showed an increase in thalamic microglia compared to WT rats and SHR-SP rats (Figures 8E–H). It is also noteworthy that the microglia adopt an activated morphology in the rTg-DI rats and bigenic rTg-DI/SHR-SP rats whereas in the WT rats and SHR-SP rats the microglia exhibit a resting surveillance state with numerous extended processes. Together, these findings indicate that the robust neuroinflammation in response to microvascular amyloid in rTg-DI rats is preserved in the bigenic rTg-DI/SHR-SP rats. Further, these findings show that glial activation is not appreciably observed with HTN alone in the SHR-SP rats.

### Hypertension Phenotype Does Not Influence the Number of Microbleeds in the Brains of 10 M Bigenic Rat Model of Cerebral Amyloid Angiopathy/Spontaneously Hypertensive, Stroke Prone Rats

**FIGURE 9:**
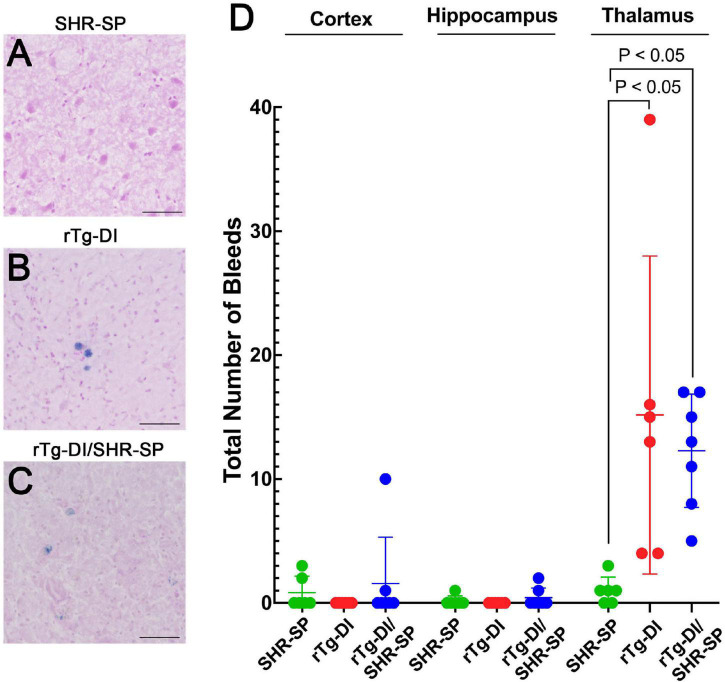
Cerebral microbleeds in 10 M rats. Representative images of the thalamic region of 10 M old SHR-SP **(A)**, rTg-DI **(B)**, and bigenic rTg-DI/SHR-SP **(C)** stained with hemosiderin to identify cerebral microbleeds. Scale bars = 50 μm. **(D)** Number of microbleeds in different brain regions are shown in SHR-SP (*n* = 6) in green, rTg-DI (*n* = 6) in red, and rTg-DI/SHR-SP (*n* = 7) in blue. Data shown are means ± S.D. Significant differences are shown by one-way ANOVA between rTg-DI and SHR-SP (*P* < 0.05) and between bigenic rTg-DI/SHR-SP and SHR-SP (*P* < 0.05). These differences indicate a preservation of the microbleeding pattern observed in rTg-DI in bigenic rTg-DI/SHR-SP animals.

Thalamic microbleeds are a prominent pathological feature of rTg-DI rats that emerge at ≈6 M of age (Davis et al., 2018). Cerebral microbleeds also occur in different brain regions of aged SHR-SP rats (Schreiber et al., 2012, 2013). Therefore, we evaluated the presence of cerebral microbleeds in the cortex, hippocampus and thalamus of all rats by performing hemosiderin staining. Figures 9A–C shows representative images of microbleeds in the thalamic region of the rats. WT rats show no evidence of microbleeds in the thalamus or any other region (data not shown). Quantitation of microbleeds in the different brain regions of the rats was performed. Kruskal Wallis test of means comparison of each positive group followed by a Dunn’s test was completed for the cortex, hippocampus and thalamus to compare the total number of bleeds in SHR-SP (*n* = 6), rTg-DI (*n* = 6), and rTg-DI/SHR-SP (*n* = 7) (Figure 9D) revealing several findings. First, in the cortex several SHR-SP and bigenic rTg-DI/SHR-SP rats showed some cerebral microbleeds whereas the rTg-DI rats did not show any. The same trend is observed for microbleeds in the hippocampus. Lastly, the thalamus is most affected with numerous microbleeds in rTg-DI and rTg-DI/SHR-SP while SHR-SP show lower levels similar to those observed in the cortex. The Kruskal-Wallis test showed significance with (*P* = 0.0004) and a Kruskal-Wallis statistic value of 11.80. Differences were found between number of bleeds in the thalamus of SHR-SP and rTg-DI (*P* < 0.05) and SHR-SP and rTg-DI/SHR-SP (*P* < 0.05). These results indicate the number of microbleeds in thalamus of rTg-DI and rTg-DI/SHR-SP are similar and higher when compared to SHR-SP rats. Thus, there is a preservation of rTg-DI thalamocentric pattern of microbleeds with addition to the hypertensive SHR-SP background.

### Hypertension Phenotype Impacts Thalamic Small Vessel Occlusions in 10 M Bigenic Rat Model of Cerebral Amyloid Angiopathy/Spontaneously Hypertensive, Stroke Prone Rats

**FIGURE 10:**
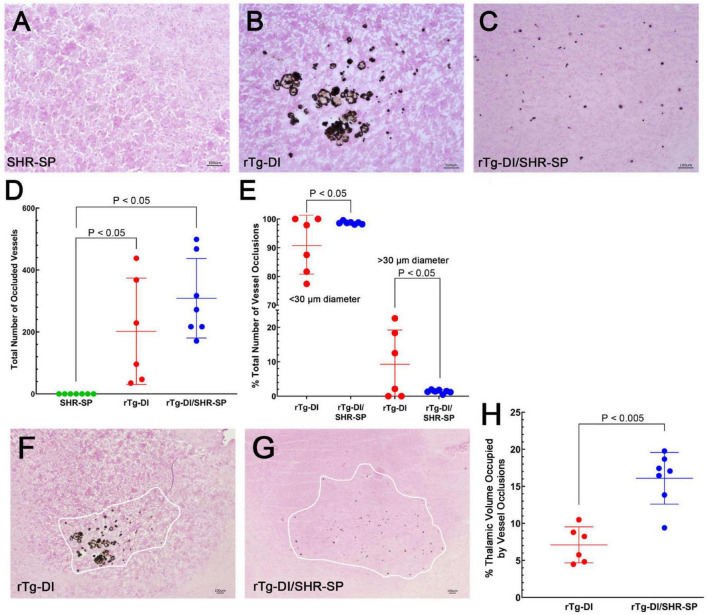
Thalamic small vessel occlusions in 10 M rats. Representative images of 10 M brain sections of SHR-SP **(A)**, rTg-DI **(B)**, and bigenic rTg-DI/SHR-SP **(C)** rats showing thalamic calcified, occluded vessels. Scale bars = 100 μm. **(D)** Total number of thalamic occluded vessels in SHR-SP (*n* = 7) in green, rTg-DI (*n* = 6) in red and bigenic rTg-DI/SHR-SP (*n* = 7) in blue **(D)**. Data presented are the means ± S.D. Significant differences were found by one-way ANOVA between SHR-SP and rTg-DI (*P* < 0.05) and between SHR-SP and bigenic rTg-DI/SHR-SP (*P* < 0.05). **(E)** The percentage of total thalamic vessel occlusions < or > 30 μm in diameter in rTg-DI (*n* = 6; red) and bigenic rTg-DI/SHR-SP (*n* = 7; blue). Data presented are the means ± S.D. Unpaired *t*-test shows an increase in the percentage of thalamic vessel occlusion diameters < 30μm or > 30 μm in bigenic rTg-DI/SHR-SP compared to rTg-DI (*P* < 0.05). Lower magnification representative images of 10 M brain sections of rTg-DI **(F)** and bigenic rTg-DI/SHR-SP **(G)** rats showing the range of thalamic calcified, occluded vessels. White tracings depict the borders of small vessel occlusions in thalamus. Scale bars = 100 μm. **(H)** The percent thalamic volume presenting with small vessel occlusions in rTg-DI (*n* = 6) in red and bigenic rTg-DI/SHR-SP (*n* = 7) in blue. Data presented are the means ± S.D. Unpaired *t-*test shows a significant increase in thalamic volume with small vessel occlusions in bigenic rTg-DI/SHR-SP rats compared to rTg-DI rats (*P* < 0.005). Together, these data indicate that the number of thalamic vessel occlusions does not change with the introduction of the rTg-DI transgene onto the SHR-SP background, although there is a change in size and extent of spatial distribution of these vessel occlusions in the thalamus.

In the rTg-DI model, calcified small vessel occlusions emerge at ≈6 M and increase with further aging (Davis et al., 2018). We next determined if the hypertensive SHR-SP background impacts the presence and numbers of these small vessel occlusions. Figure 10 shows representative images of calcified, occluded small vessels in the thalamic region of the different rats (Figures 10A–C). Occluded vessels were observed only in the thalamus of rTg-DI and bigenic rTg-DI/SHR-SP rats. Kruskal Wallis test of means was completed for the total number of occluded vessels in the thalamus of SHR-SP, rTg-DI, and bigenic rTg-DI/SHR-SP (Figure 10D). Significant differences were found between SHR-SP rats and rTg-DI and bigenic rTg-DI/SHR-SP (*P* < 0.05) since SHR-SP itself showed no vessel occlusions. Though the total number of occlusions did not differ between rTg-DI and bigenic rTg-DI/SHR-SP animals, a qualitative difference was observed in the sizes and spatial distribution of the occlusions (Figures 10B,C). We next measured the diameters of occlusions and sorted them based on size. The number of small and large occlusions are shown as percentage of total number of vessel occlusions in the thalamus (Figure 10E). Unpaired *t*-test shows an increase in the percentage of small vessel occlusions in bigenic rTg-DI/SHR-SP thalamus compared to rTg-DI thalamus (*P* < 0.05). On the other hand, a significant increase was found in the percentage of larger occlusions in the thalamus of rTg-DI rats compared to bigenic rTg-DI/SHR-SP rats (*P* < 0.05). A difference was also observed in the percent of thalamic volume that presented small vessel occlusions between rTg-DI and bigenic rTg-DI/SHR-SP rats by two-tailed, unpaired parametric *t*-test (*P* < 0.005) (Figures 10F–H). These findings show that even though the total number of small vessel occlusions did not differ between rTg-DI rats and bigenic rTg-DI/SHR-SP rats the latter exhibited primarily small occlusions that were more broadly distributed in the thalamus.

### Elevated Cortical Proteins in Spontaneously Hypertensive, Stroke Prone, Rat Model of Cerebral Amyloid Angiopathy and Bigenic Rat Model of Cerebral Amyloid Angiopathy/Spontaneously Hypertensive, Stroke Prone

**FIGURE 11:**
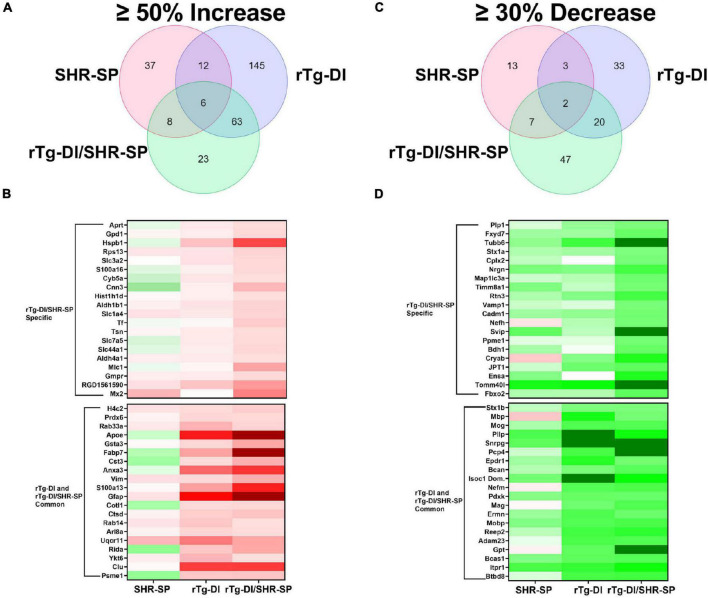
Comparison of significantly enhanced and reduced proteins in the 10 M SHR-SP, rTg-DI and bigenic rTg-DI/SHR-SP cortex. **(A)** Venn diagram comparing significantly (*P* < 0.05) enhanced proteins by ≥ 50% of the WT concentration in the cortex of 10 M SHR-SP, rTg-DI and bigenic rTg-DI/SHR-SP rats (*n* = 5, 6). **(B)** Heat map depicting relative expression of most abundantly enhanced cortical proteins in the bigenic rTg-DI/SHR-SP only (top) or common to both rTg-DI and bigenic rTg-DI/SHR-SP, along with the corresponding expression in the SHR-SP cortex, with red indicating enhanced expression, green representing reduced expression, and color intensity representing the degree of expression change. **(C)** Venn diagram comparing significantly (*P* < 0.05) reduced proteins by ≥ 30% of the WT concentration in the cortex of 10 M SHR-SP, rTg-DI and bigenic rTg-DI/SHR-SP rats (*n* = 5, 6). **(D)** Heat map depicting relative expression of most reduced proteins arranged as in **(B)** with color shading as in **(B)**.

To further understand the impact of the SHR-SP hypertensive phenotype on the pathology of the rTg-DI rats we conducted proteomic analysis of cortical tissue from WT, SHR-SP, rTg-DI, and bigenic rTg-DI/SHR-SP rats via Sequential Acquisition of all Theoretical Mass Spectra (SWATH-MS), a data independent acquisition (DIA) protein mass spectrometry approach, as previously described (Schrader et al., 2021) and in section “Materials and Methods.” Protein identification and quantification from DIA data was performed using Spectronaut (Biognosys), referencing a previously compiled spectral library (Schrader et al., 2021). Protein intensities from Spectronaut were converted to molar concentrations using the “total protein approach” (TPA) (Wiśniewski and Rakus, 2014). Differentially expressed proteins were then determined by comparison of molar concentrations (pmol/mg total protein) with the corresponding WT concentrations. As previously reported, multiple testing corrected false discovery rates (FDR) are often too restrictive in small *n* proteomics studies (Pascovici et al., 2016; Hondius et al., 2018), and thus we used threshold cutoffs with uncorrected *P-*values to manage the FDR as before (Schrader et al., 2021). Significantly increased proteins were defined as ≥ 50% increase with *P* ≤ 0.05. From this analysis 63, 226, and 100 proteins were found significantly elevated in the cortex of the SHR-SP, rTg-DI, and bigenic rTg-DI/SHR-SP rats, respectively (Figure 11A), and lists of these proteins can be found in Supplementary Tables 1–3. The rTg-DI and bigenic rTg-DI/SHR-SP rats displayed the greatest commonality, as a total of 69 elevated proteins were shared between the two, whereas only 6 proteins were common to all three models. The bigenic rTg-DI/SHR-SP and SHR-SP models shared only 14 elevated proteins (Figure 11A). Heat maps depicting the relative expression of the 20 most abundant elevated proteins in the bigenic rTg-DI/SHR-SP rats along with a heat map depicting the 20 most abundant elevated proteins common to the rTg-DI and bigenic rTg-DI/SHR-SP rats is shown in Figure 11B, including the corresponding expression in the SHR-SP cortex. Red shading indicates increased, green decreased, and white no change in expression, with color intensity relative to the degree of change. Of note among these proteins is Hspb1, which is strongly enhanced in bigenic rTg-DI/SHR-SP cortex, but not in that of the other models. We have previously reported upregulation of Hspb1 other brain regions of rTg-DI rats that display greater CAA burden and more severe CAA related pathology (Schrader et al., 2021). Furthermore, many of the significantly enhanced proteins shared by the rTg-DI and bigenic rTg-DI/SHR-SP rats display greater increases in the bigenic rTg-DI/SHR-SP cortex. For example, Apoe, Gsta3, Fabp7, Cst3, Anxa3, Gfap, and S100a13 were all previously reported as commonly elevated in the cortex, thalamus, and hippocampus of rTg-DI rats (Schrader et al., 2021). All of these proteins are commonly elevated in the rTg-DI and bigenic rTg-DI/SHR-SP cortex (Figure 11B), but to a greater extent in the bigenic rTg-DI/SHR-SP animals. Taken together, this could suggest an enhancing effect of the SHR-SP hypertensive phenotype on the rTg-DI model, as proteins that were elevated in the cortex of rTg-DI rats are elevated to a greater degree in the bigenic rTg-DI/SHR-SP rats. In addition, Anxa3, Vim, Clu, and Ctsd, all previously reported to be elevated in brain regions of 12 M rTg-DI rats, were commonly elevated in the 10 M rTg-DI and bigenic rTg-DI/SHR-SP cortex. Of particular note is Anxa3, which has been previously suggested as a marker of microglia activation (Junker et al., 2007; Smithson and Kawaja, 2010), and proven to be an indicator of microgliosis in rTg-DI rats (Schrader et al., 2021). The finding that many of these proteins are not elevated in the SHR-SP cortex further suggests that the presence of CAA is responsible for their consistent expression profile between rTg-DI and bigenic rTg-DI/SHR-SP rats.

### Reduced Cortical Proteins in Spontaneously Hypertensive, Stroke Prone, Rat Model of Cerebral Amyloid Angiopathy and Bigenic Rat Model of Cerebral Amyloid Angiopathy/Spontaneously Hypertensive, Stroke Prone Rats

We also compared the significantly reduced (≥30% decrease, *P* ≤ 0.05) proteins in the cortex of each model (Figures 11C,D). In this comparison 25, 58, and 76 proteins were decreased in the SHR-SP, rTg-DI, and bigenic rTg-DI/SHR-SP cortex, respectively, and lists of these proteins can be found in Supplementary Tables 4–6. Again, the rTg-DI and bigenic rTg-DI/SHR-SP rats shared the greatest commonality, as 22 reduced proteins were shared between the two, whereas only 9 were shared between the SHR-SP and bigenic rTg-DI/SHR-SP rats (Figure 11C). Notable among the shared proteins are myelin oligodendrocyte glycoprotein (Mog), myelin associated glycoprotein (Mag), myelin basic protein (Mbp), myelin oligodendrocyte basic protein (Mobp), and neurofilament medium polypeptide (Nefm). Mog, Mag, Mbp, and Mobp (Yoshikawa, 2001; Pronker et al., 2016; Weil et al., 2016; Peschl et al., 2017). All contribute to axonal myelination, and thus significant reduction in their expression could lead to demyelination and disruption of axonal integrity, while alteration in neurofilament expression is often indicative of neurodegeneration (Liu et al., 2011). These results are consistent with the diffuse white matter loss previously reported in brain regions of the rTg-DI rats, along with our previous findings of their differential expression (Lee et al., 2021; Schrader et al., 2021). Proteolipid protein 1 (Plp1) and neurofilament heavy polypeptide (Nefh), listed as specifically down regulated in the bigenic rTg-DI/SHR-SP cortex, did not meet our threshold cutoffs in the rTg-DI cortex, though their 25 and 18% respective decreases were statistically significant. Considering their implicated roles in myelination (Plp1) (Gould et al., 2018) and axonal integrity (both) (Liu et al., 2011; Gould et al., 2018), this is consistent with our other findings. In any case, like the upregulated proteins, the observed trends in the down regulated proteins suggest that the bigenic rTg-DI/SHR-SP rats adopt a proteome much more similar to the rTg-DI rats than the SHR-SP.

### Pathway Analysis of Differentially Expressed Cortical Proteins in Spontaneously Hypertensive, Stroke Prone, Rat Model of Cerebral Amyloid Angiopathy and Bigenic Rat Model of Cerebral Amyloid Angiopathy/Spontaneously Hypertensive, Stroke Prone Rats

**FIGURE 12:**
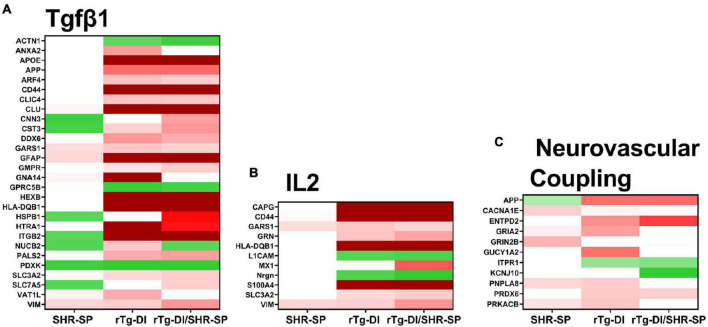
IPA identified upstream regulators, causal networks, and disease functions. **(A)** Heat map depicting the differentially expressed proteins (≥ 50% increase or ≥ 30% decrease, *P* < 0.05) in the SHR-SP, rTg-DI, and bigenic rTg-DI/SHR-SP cortex associated with the upstream regulator Tgfβ1 **(A)**, Interleukin 2 causal network **(B)** and the pathway neurovascular coupling **(C)**. Red indicates increased, green indicates decreased, and white indicates not differentially expressed proteins, and color intensity correlates with degree of change.

To provide functional context to the similarities and differences observed in the different rat model proteomes, we performed comparative pathway analysis using Ingenuity Pathway Analysis (IPA) (QIAGEN Inc.).^1^ Only proteins meeting our imposed threshold cutoffs were included in the analysis. IPA predicts activation (z score > 2) or inhibition (z score < −2) states of upstream regulators, causal networks, or disease functions based on the directional differential expression of downstream or associated target proteins (Krämer et al., 2014). Comparative analysis predicted activation of the upstream regulator TGF-β1 in the rTg-DI and bigenic rTg-DI/SHR-SP cortex, but not in the SHR-SP. This is consistent with our previous reports of increased mRNA expression of TGF-β1 in the brain of rTg-DI rats and IPA predicted activation of TGF-β1 in brain regions of 12 M rTg-DI rats (Zhu et al., 2020; Schrader et al., 2021). A heat map comparing relative fold changes in each model of downstream proteins associated with TGF-β1 is displayed in Figure 12A, with red color indicating increased expression, green decreased, and color intensities relative to the degree of change. Proteins that did not meet the imposed effect threshold cutoffs are depicted in white as they were not considered in the IPA analysis. Many of the depicted proteins, such as Apoe, App, clusterin (Clu), Gfap, serine protease Htra1 (Htra1), vimentin (Vim), and integrin β-2 (IGB2) are not only common to rTg-DI and bigenic rTg-DI/SHR-SP cortex, but were also previously found to be elevated in other brain regions of 12 M rTg-DI rats (Schrader et al., 2021). Also noteworthy is Hspb1, which, as stated above, is specifically upregulated in the bigenic rTg-DI/SHR-SP cortex, but not in the rTg-DI nor the SHR-SP cortex.

IPA also indicated activation of Interleukin 2 (IL2) in the bigenic rTg-DI/SHR-SP cortex, but not in that of the SHR-SP. Only 2 of the 11 proteins were somewhat enhanced in the SHR-SP cortex. A heat map comparing relative fold changes in each model of downstream proteins associated with IL2 is depicted in Figure 12B, with color shading as above. IL2 has been reported to enhance astrocyte recruitment and activation of astrocytes and lead to decreased amyloid load in the mouse hippocampus (Alves et al., 2017). Additionally, IL2 has been shown to influence activation of macrophages by directly mediating the release of TGF-β1 (Nelson et al., 1994). On the other hand, IL2 also disrupts BBB integrity and can lead to vascular leak syndrome (Wylezinski and Hawiger, 2016), which could exacerbate microbleeds. Interestingly, many of the proteins indicated here for bigenic rTg-DI/SHR-SP rats are also differentially expressed in the rTg-DI cortex, though it did not meet the threshold (z score > 2) for predicted activation. Thus, these results, along with the activation of TGF-β1 mentioned above, further suggest that the bigenic rTg-DI/SHR-SP rats closely resemble that of the rTg-DI rats, where the SHR-SP hypertensive phenotype exacerbates changes in the cortex.

IPA also predicted activation of the Neurovascular Coupling pathway in the cortex of rTg-DI rats, but not the bigenic rTg-DI/SHR-SP nor the SHR-SP cortex (Figure 12C). Neural vascular coupling is the mechanism responsible for altering localized cerebral blood flow in response to enhanced neuronal activity (Hendrikx et al., 2019). Neurovascular coupling will regionally enhance cerebral blood flow to areas of enhanced neuronal activity to support neuronal function while aiding in waste removal (Kaplan et al., 2020). Considering its role in removal of waste, neurovascular coupling may be important for amyloid clearance. Although some of these features are lost in the bigenic rTg-DI/SHR-SP cortex it still more closely resembles rTg-DI cortex rather than SHR-SP.

## Discussion

The impact of HTN on CAA and ICH remains controversial. Clinically, ICH in CAA patients occurs mostly in the cortex of elderly patients (Sahni and Weinberger, 2007; Mehndiratta et al., 2012). CAA patients have a lower mortality rate but higher incidence of recurrence than HTN patients (Mehndiratta et al., 2012). On the other hand, HTN generally affects younger patients and associated bleeds typically occur in deeper regions of the brain, particularly the basal ganglia, cerebellum and pons (Sutherland and Auer, 2006; Sahni and Weinberger, 2007). A clinical study of risk of ICH showed that patients diagnosed with both HTN and CAA had lower incidences of ICH (mixed ICH) compared to patients with only CAA (Pasi et al., 2018). On the other hand, mixed ICH patients seemed to have a higher incidence of ICH than only HTN ICH occurrences (Pasi et al., 2018) suggesting a possible protective effect of HTN in CAA. Another study reported similar results; CAA patients with HTN had better clinical outcomes after ICH events (Zhang et al., 2020). Treating with anti-hypertensive therapies was shown to reduce incidence of ICH and stroke in HTN patients (Qureshi et al., 2001) and was also found to decrease ICH incidence in CAA patients (Arima et al., 2010). Overall, clinical data suggests that HTN and CAA as comorbidities could provide a protective effect against characteristic ICH events of CAA but further investigation is required.

There have been previous experimental studies on the effects of acute and chronic HTN in AD and CAA in murine models. For example, it was reported that AD-like symptoms in Tg-SwDI mice worsened with pharmacologically induced chronic HTN (Kruyer et al., 2015). In this study, the endothelial nitric oxide synthase (eNOS) inhibitor, L-NAME (Wu et al., 2020) was used. In similar studies, HTN was pharmacologically induced by eNOS inhibition in conjunction with administration of angiotensin II in the Tg2576 mouse model of AD-like pathology (Passos et al., 2016; Nyúl-Tóth et al., 2020). In both cases, there was an increase in cerebral microbleeds. However, the inhibition of eNOS can be controversial as this has been shown to cause an increase in AβPP expression in mice (Austin et al., 2013) and pharmacological inhibitors can target other NOS isoforms (Alderton et al., 2001). In another study HTN was induced in mice by transverse aortic coarctation resulting in brain injury in the cortex and hippocampus and increased Aβ in brain (Carnevale et al., 2012). Although this is not a pharmacological intervention, transverse aortic coarctation is used to induce heart failure and could cause excessive cardiac effects with undesired consequences (deAlmeida et al., 2010).

In the present study, we investigate how chronic, non-pharmacological HTN interacts as a comorbidity of CAA, a prominent amyloidal CSVD and cause of stroke, using novel rat models of disease. There are several advantages to our approach to study these interactions. First, in contrast to the studies mentioned above that used murine models, here we use rat models of CAA and HTN. Rat models may be more suited for investigating human disease. In particular, the rTg-DI rat model of CAA faithfully recapitulates many of the pathological features of clinical CAA including perivascular neuroinflammation, cerebral microbleeds, small vessel occlusions, progressive white matter loss and progressive behavioral decline (Davis et al., 2018; Popescu et al., 2020; Zhu et al., 2020; Lee et al., 2021). Second, the use of rTg-DI rats focuses specifically on CAA pathology without parenchymal AD-like pathologies observed in most murine models. Lastly, due to the genetic origin of the phenotype, the use of a spontaneous, non-pharmacological rat model of HTN eliminates the potential confounds of any pharmacological or surgical side effects. In this regard, the SHR-SP model is appropriate due to its clinically relevant pathologies such as patterns of bleeding (Yamori et al., 1976). In addition, both the SHR-SP and rTg-DI models have consistent timelines of emerging pathology. SHR-SP rats are shown to develop and maintain hypertensive systolic blood pressure starting at ≈10 weeks of age (Okamoto and Aoki, 1963). This is harmonious with the rTg-DI rats that begin to accumulate CAA at ≈3 M of age (Davis et al., 2018; Zhu et al., 2020). The reliable timing of both models produces a cross that steadily exhibits clinically relevant pathologies of both CSVDs.

Crossbreeding of the SHR-SP rats and rTg-DI rats showed a preservation of both model phenotypes. Systolic blood pressures of SHR-SP rats and bigenic rTg-DI/SHR-SP rats were increased compared to WT rats and rTg-DI rats at 7 M. Despite the elevated blood pressures in bigenic rTg-DI/SHR-SP rats this had no appreciable impact on the level of transgene human AβPP expression or in the accumulation of Aβ peptides in the brain at 7 M (Figures 2, 3). HTN at this age also did not affect cognitive decline characteristic of rTg-DI rats. Because 7 M bigenic rTg-DI/SHR-SP rats showed no changes from rTg-DI other than increased systolic blood pressure, we bred a second cohort of animals that were aged to 10 M to determine the effects of HTN in rTg-DI rats that exhibit more advanced pathologies.

In addition to already increased systolic pressure observed in 7 M animals, SHR-SP and bigenic rTg-DI/SHR-SP rats at 10 M also exhibited increased diastolic blood pressures compared to WT and rTg-DI rats. Though AβPP expression (not shown) and Aβ peptide accumulation were not different between 10 M rTg-DI and bigenic rTg-DI/SHR-SP animals the SHR-SP background was correlated with a significant redistribution of CAA load in the bigenic rTg-DI/SHR-SP rats. The thalamic and hippocampal vascular amyloid loads of the bigenic rTg-DI/SHR-SP rats were significantly decreased whereas the surface pial vessel amyloid load was more than doubled in the same rats indicating a significant shift in amyloid distribution to a different vascular bed. Though the microvascular CAA load in the cortex was not significantly different, there appears to be a modest increase in the rTg-DI/SHR-SP rats and that could indicate an emerging change. It was previously reported that CAA spontaneously develops in SHR-SP rats (Carnevale et al., 2012; Jandke et al., 2018; Denver et al., 2019). However, we were unable to detect the accumulation of vascular Aβ or fibrillar amyloid in the 10 M SHR-SP rats used in this study. This redistribution of vascular amyloid load in bigenic rTg-DI/SHR-SP rats could result from different effects of HTN. For example, HTN is known to lower cerebral vasoreactivity (Hajjar et al., 2010). Also, cerebrospinal fluid (CSF) flow is driven by arterial pulsations and is reduced in HTN (Mestre et al., 2018). In this regard, we recently found that rTg-DI rats exhibit a hyperdynamic CSF flow coupled with reduced glymphatic clearance compared with WT rats (Chen et al., 2022). In future studies it will be interesting to investigate if the HTN phenotype impacts CSF flow and glymphatic clearance in bigenic rTg-DI/SHR-SP rats.

The rTg-DI rats typically present with microbleeds that are largely restricted to the thalamus and emerge at ≈6 M of age (Davis et al., 2018). SHR-SP animals were reported to exhibit microbleeds emerging at ≈3 M which increase in severity and number with age (Schreiber et al., 2012). These previous findings in the CAA and HTN rat models are consistent with clinical ICH where CAA bleeds affect more elderly individuals whereas HTN patients with bleeds are generally younger (Sahni and Weinberger, 2007; Mehndiratta et al., 2012). In our study, SHR-SP rats showed some microbleeds in all brain regions whereas in rTg-DI rats they were mostly observed in the thalamic region. The presence of HTN in bigenic rTg-DI/SHR-SP rats appears to somewhat enhance the number of microbleeds in the thalamus, although no significant difference was observed.

Similarly, small vessel thalamic occlusions also emerge at ≈6 M (Davis et al., 2018). SHR-SP rats showed no small vessel occlusions in the thalamus or in any other brain region. It should be noted that infarcts occurring in SHR-SP animals are associated with blood-brain barrier (BBB) compromise rather than vessel occlusions (Schreiber et al., 2013), supporting the lack of vessel occlusions found in the SHR-SP rats. Although there was no significant difference found between the number of small vessel occlusions in the thalamus of rTg-DI rats and bigenic rTg-DI/SHR-SP rats, a clear qualitative difference was observed in the size and area occupied by vessel occlusions (Figures 10A–C). Further quantitation of size and spatial distribution of occlusions confirmed that there is a change in the characteristics of vessel occlusions when rTg-DI rats are on the SHR-SP background. Bigenic rTg-DI/SHR-SP rats have smaller and more dispersed occlusions in the thalamus than rTg-DI rats. It has been reported in several studies that blood vessel lumens decrease in diameter due to contraction of the blood vessels, thickening of vessel walls, and overall change in function of vessels as a result of HTN (Schiffrin, 1992; Intengan and Schiffrin, 2000; Mulvany, 2002; Pires et al., 2013). Studies of SHR-SP rats have shown that remodeling of cerebral arterioles occurs in older (6–10 M) rats and is characterized by thickening of the blood vessel wall and decrease of lumen diameter (Baumbach et al., 1988; Baumbach and Heistad, 1989). This HTN decrease in vessel lumens could physically be preventing the characteristic larger occlusions observed in rTg-DI rats from forming in bigenic rats. It is also possible that the global remodeling of arterioles caused by HTN is impacting the area affected within the thalamus leading to the wider distribution of occluded vessels in bigenic rats, although this would need further study.

Proteomic analysis of the SHR-SP, rTg-DI and bigenic rTg-DI/SHR-SP cortex revealed much greater similarity between the rTg-DI and bigenic rTg-DI/SHR-SP models, as they shared 91 differentially expressed proteins compared to only 23 common differentially expressed proteins between the SHR-SP and bigenic rTg-DI/SHR-SP cortex (Figures 11A,C). Many of the commonly reduced proteins including Mbp, Mobp, Mog, Mag, and Nefm, are associated with myelination, axonal integrity, and neuronal degeneration. Thus, these results are consistent with our previous findings of diffuse white matter loss and down regulation of these proteins in similarly aged rTg-DI rat brains (Lee et al., 2021; Schrader et al., 2021). Notable among the commonly elevated proteins are Apoe, Anxa3, and Gfap. We have previously reported elevation of Apoe in brain regions of 12 M rTg-DI rats with strong co-localization of Apoe and vascular amyloid deposits (Schrader et al., 2021). Thus, elevated Apoe in the bigenic rTg-DI/SHR-SP cortex is not surprising due to the abundant vascular amyloid in these animals. It is not surprising that Gfap, a well-known astrocyte marker, is robustly elevated in both bigenic rTg-DI/SHR-SP and rTg-DI rats since these animals similarly present with increased astrocytes (Figure 8; Davis et al., 2018; Zhu et al., 2020; Schrader et al., 2021). Similarly, we previously reported Anxa3, a marker of activated microglia, is elevated in rTg-DI rats (Schrader et al., 2021). Thus, elevated levels of Anax3 in bigenic rTg-DI/SHR-SP rats is consistent with increased microglial activation observed in both rTg-DI rats and bigenic rTg-DI/SHR-SP rats (Figure 8). Taken together, these results suggest that bigenic rTg-DI/SHR-SP rats adopts a proteome more similar to the rTg-DI rats than the SHR-SP rats, and the SHR-SP hypertensive phenotype may enhance the differential expression of many of these shared proteins.

IPA analysis of the proteomes obtained from the three models predicted common and distinct activation of pathways and regulators related to inflammation, BBB integrity/permeability, and changes in cerebral blood flow. Activation of TGF-β1 was predicted in the cortex of rTg-DI and rTg-DI/SHR-SP rats (Figure 12A). We have previously shown increases in TGF-β1 mRNA expression and IPA indicated activation of TGF-β1 in 12 M rTg-DI rats, and other studies have linked upregulation of TGF-β1 mRNA in Dutch-type CAA to increased CAA severity (Moursel et al., 2018; Zhang and Yang, 2020; Zhu et al., 2020; Schrader et al., 2021). Interestingly, it has been reported that TGF-β1 deficiency in the neurovascular unit increases BBB permeability and that TGF-β1 released from astrocytes promotes BBB integrity (Garcia et al., 2004; Derada Troletti et al., 2016). Considering these reported roles of TGF-β1 in the neurovascular unit, it is interesting that the neurovascular coupling pathway was only predicted to be activated in the rTg-DI cortex (Figure 12C). Neurovascular coupling, mediated by vascular smooth muscle, astrocytes and neurons, is responsible for regional changes in cerebral blood flow in response to neuronal activity (Hendrikx et al., 2019; Kaplan et al., 2020). These changes in blood flow promote essential nutrient delivery and waste removal (Kaplan et al., 2020), and therefore may be important for Aβ clearance. Thus, the lack of activation of neurovascular coupling in the bigenic rTg-DI/SHR-SP cortex could suggest altered deposition or clearance of vascular amyloid, and may lead to changes in CAA pathology, however, this requires further investigation. IPA predicted activation of IL2 only in the bigenic rTg-DI/SHR-SP rats, although 10 of the 11 implicated proteins were also differentially expressed in the rTg-DI rats. IL2 activity has been reported to promote clearance of Aβ through the activation and recruitment of astrocytes (Alves et al., 2017). On the other hand, IL2 may be detrimental to BBB integrity and can lead to vascular leak syndrome (Wylezinski and Hawiger, 2016). Thus, IL2 could have beneficial or damaging functions in CAA related pathology depending on its context. Nevertheless, the differential expression of proteins associated with IL2 activation further suggests that the bigenic rTg-DI/SHR-SP proteome is most closely related to the rTg-DI model, and may not only enhance these protein changes, but the activation of pathways as well.

Despite the new information gained there are several noted limitations of the present study. Our investigations focused primarily on 10 M old rats where CAA and associated vascular pathologies are developed but, still progressing. Potential chronic effects of HTN at this stage of disease may not be prominent but could be become much more robust as the animals continue to age and disease progresses. For example, a recent study similarly bred SHR-SP rats with a rat model of AD-like pathologies (Denver et al., 2019). Aging of these particular bigenic animals to 16–18 M showed a worsening of several AD-like pathologies in the brain including vascular changes, neuroinflammation and mitochondrial stress, but no effect on behavioral deficits (Denver et al., 2019). This underscores how further aging of our bigenic rTg-DI/SHR-SP rats may be necessary to observe more robust effects of HTN on CAA related pathologies. Second, the rTg-DI rat is a model of CAA type-1 that primarily affects small microvessels and capillaries. Perhaps HTN introduced by the SHR-SP crossing would be more impactful on a model of CAA type-2 that targets larger vessels in the brain. Finally, the rTg-DI model involves cerebral vascular deposition of chimeric Dutch/Iowa familial CAA mutant Aβ. HTN could have a more prominent impact on CAA accumulation and associated pathologies in a model that accumulates non-mutated Aβ. In any case, the changes caused by HTN that are observed in this study suggest that over time, the CAA pathologies could be further altered and perhaps have a significant impact on cognitive deficits. The hypertensive bigenic rTg-DI/SHR-SP rats generated in this study provide a preclinical platform to further investigate the consequences of chronic, non-pharmacological HTN on CAA and VCID.

## Data Availability Statement

Raw mass spectrometry data can be found in the MassIVE repository (massive.ucsd.edu/ProteoSAFe/static/massive.jsp), project ID#: MSV000088300, and password: SvdCortComp21.

## Ethics Statement

The animal study was reviewed and approved by the University of Rhode Island Institutional Animal Care and Use Committee.

## Author Contributions

AS performed experiments, analyzed the data, and wrote the manuscript. JS performed the experiments, analyzed the data, and edited the manuscript. XZ, JM, MM, and FX performed the experiments and analyzed the data. AH performed the experiments. JR designed the experiments and analyzed the data. WV conceived the study, designed the experiments, edited the manuscript, and secured funding. All authors contributed to the article and approved the submitted version.

## Conflict of Interest

The authors declare that the research was conducted in the absence of any commercial or financial relationships that could be construed as a potential conflict of interest.

## Publisher’s Note

All claims expressed in this article are solely those of the authors and do not necessarily represent those of their affiliated organizations, or those of the publisher, the editors and the reviewers. Any product that may be evaluated in this article, or claim that may be made by its manufacturer, is not guaranteed or endorsed by the publisher.

## Footnotes

1
massive.ucsd.edu/ProteoSAFe/static/massive.jsp
